# Antioxidant Effects of a Polyphenol-Rich Dietary Supplement Incorporating *Pinus massoniana* Bark Extract in Healthy Older Adults: A Two-Arm, Parallel Group, Randomized Placebo-Controlled Trial

**DOI:** 10.3390/antiox11081560

**Published:** 2022-08-11

**Authors:** Jessica J. A. Ferguson, Christopher Oldmeadow, David Bentley, Manohar L. Garg

**Affiliations:** 1 Nutraceuticals Research Program, School of Biomedical Sciences & Pharmacy, University of Newcastle, Callaghan, NSW 2308, Australia; 2 Clinical Research Design, Information Technology and Statistical Support Unit, Hunter Medical Research Institute, University of Newcastle, New Lambton, NSW 2308, Australia; 3 School of Life and Environmental Science, University of Newcastle, Ourimbah, NSW 2258, Australia

**Keywords:** ageing, pine bark, proanthocyanidins, antioxidants, polyphenols, oxidative stress

## Abstract

Oxidative stress is a key physiological phenomenon underpinning the ageing process and plays a major developmental role in age-associated chronic diseases. This study investigated the antioxidant effects of a polyphenol-rich dietary supplement containing *Pinus massoniana* bark extract (PMBE) in healthy older adults. In a double-blinded, placebo-controlled clinical trial, participants were randomised (in a 1:1 ratio) to receive a 50 mL/day dietary supplement containing placebo (0 mg PMBE) or PMBE (1322 mg PMBE) for 12 weeks. The primary outcome was fasting plasma malondialdehyde (MDA) concentrations and secondary outcomes were plasma inflammatory markers. MDA concentrations significantly reduced following PMBE for 6 weeks (−1.19 nmol/mL, 95%CI −1.62, −0.75, *p* < 0.001) and 12 weeks (−1.35 nmol/mL, 95%CI −1.74, −0.96, *p* < 0.001) compared to baseline. MDA did not significantly change after the placebo. MDA levels at 6 and 12 weeks were significantly lower following PMBE compared to placebo (*p* < 0.001). At 12 weeks in the PMBE group, fibrinogen concentrations significantly reduced (−0.25 g/L, 95%CI −0.39, −0.11; *p* < 0.0001) and interleukin-6 significantly increased compared to placebo (0.30 pg/mL, 95%CI 0.02, 0.59; *p* < 0.05). PMBE in a polyphenol-rich dietary supplement reduced oxidative stress in healthy older adults. Further studies are warranted to investigate the antioxidant capacity of PMBE in conditions with heightened oxidative stress, such as osteoarthritis, hypertension, type 2 diabetes, or other lifestyle related diseases.

## 1. Introduction

Australia has an increasingly ageing population, with over 27% (6.8 million) of the total population aged 55 and over in 2019 [1] and an average life expectancy now exceeding 80 years for both males and females, one of the highest in the world [2]. With such a growing older population, it is imperative to focus not only on longevity but on increasing quality of life by minimising the risk of developing age-related chronic diseases. Ageing is associated with complex changes and dysregulation of the immune system, increased low-grade chronic inflammation [3], and an imbalance between reactive oxygen/nitrogen species production and antioxidant defence [4]. The characteristic of increased concentrations of low-grade chronic inflammatory markers in the blood is a phenomenon termed ‘inflammageing’ and possesses bidirectional interactions with oxidative stress which trigger or facilitate the onset of key age-related chronic diseases, such as cardiovascular disease (CVD), type 2 diabetes and sarcopenia [3].

Up-regulation of oxidative metabolism and subsequent accumulation of abnormal reactive oxygen species (ROS) [5] leads to higher rates of cell damage including muscle damage and have been shown to modulate skeletal muscle contraction by acting on the functional status of Ca^2+^ channels [6]. Many biomarkers of oxidative stress are used, with malondialdehyde (MDA) being the most popular and reliable marker to determine oxidative stress in clinical situations as well as indicating the antioxidant capacity of certain therapies [7]. It is well known that MDA concentrations increase with increasing age [8], being highest in the elderly compared to young adults [8,9]. Given the important role of oxidative stress in the pathogenesis of several age-associated clinical conditions, antioxidant therapies may have a positive impact on the manifestation of several diseases and may even lead to enhanced quality of life throughout the progressive ageing process.

A healthy lifestyle including a balanced diet that focuses on adequate protein intake, long-term resistance/balance exercise training and some pharmacological interventions are the current preventative and therapeutic strategies for sarcopenia [10], a key clinical manifestation of ageing underpinned by increasing oxidative stress. However, long-term adherence to complex diet and lifestyle changes including those that are physically demanding can be a barrier for these individuals [11]. The addition of a safe, efficacious, adjunct therapy with potentially multiple biological targets may optimize the healthy ageing process and well-being of older adults, to lower oxidative stress and thus delay the onset of age-associated illnesses and mitigate the severity of physical symptoms, resulting in improved quality of life and lower risk of chronic disease and mortality.

Proanthocyanidins (PACs) are a structurally complex subclass of polyphenolic compounds that are widely abundant in plants. PACs are polymers of flavan-3-ols and are also known as condensed tannins [12,13] with catechin and epicatechin as the key building blocks [14]. PACs wide range of protective health benefits, such as antioxidant [15,16,17], anti-inflammatory [18,19], anticarcinogenic [20], antiviral [15], cardio-protective [21], hypotensive [22] are largely attributed to their free radical scavenging capacity and ability to inhibit lipid peroxidation. Rich dietary sources of PACs include fruits, berries, beans, nuts, cocoa, grapeseed and red wine and are responsible for the astringent and bitter flavour compounds of these foods [23]. PACs are also richly present in bark and bark extracts, with the most widely studied being the French Maritime pine (*Pinus pinaster*) in the form of dietary supplement, Pycnogenol^®^. Several human clinical studies to date have reported therapeutic applications of Pycnogenol^®^ for cardiovascular ailments and risk factors, metabolic disorders, chronic inflammatory diseases, such as asthma, type 2 diabetes and hypertension; however, there remains conflicting findings and further double-blinded clinical studies are required to provide more information on their clinical efficacy, safety and optimal dose and duration [24,25,26]. Similarly extract from the *Pinus brutia* bark has shown the potential to reduce oxidative damage due to its high free radical scavenging and 15-lipoxygenase inhibitory effects [27].

*Pinus massoniana* (PM) Lamb is derived from the south and southwest of China. Its bark, pollen, turpentine, and needles have been used in traditional Chinese medicine for the treatment of rheumatic arthralgia, hypertension, neurasthenia and chilblain [28,29]. In preclinical studies, PM bark extract (PMBE) has been demonstrated as a potential anti-metastasis agent for cancer therapy [30] and inhibited the growth of human breast cancer cells [31]. Preclinically, PMBE exerts antioxidant effects, with PMBE treatment leading to reductions in MDA and increases in glutathione (GSH), catalase, glutathione peroxidase, and superoxide dismutase (SOD), as well as alleviation of damage induced by carbon tetrachloride [32]. There are currently no human studies demonstrating the effect of PACs derived from PMBE on markers of oxidative stress. Nor are there any studies investigating the physiological effects of a dietary supplement containing PMBE. The present study aims to evaluate the effects of a polyphenol-rich dietary supplement containing PMBE on MDA concentrations as a biomarker of oxidative stress in healthy older adults.

## 2. Materials and Methods

### 2.1. Recruitment

Participants were recruited from the Hunter region (NSW, Australia) via notice board flyers placed around the local community, word of mouth, radio announcements, newspaper articles, and subjects who participated in earlier studies at our research facility were also invited to participate. Volunteers were assessed for eligibility over the phone or in person and were eligible if they were: healthy older adults aged 55–75 years old. Volunteers were ineligible if they had: a diagnosed chronic disease, such as CVD, diabetes mellitus, renal or hepatic condition, neurological condition, autoimmune condition; diagnosed chronic inflammatory condition; history of gastrointestinal disorders; or currently taking medications known to influence the study outcomes, e.g., non-steroidal anti-inflammatory medications; routinely taking supplements known to influence the study outcomes, e.g., curcumin, coenzyme Q10 or Vitamin E; taking anticoagulant medications; current smokers or smoked in the past 6 months; currently participating in another diet/lifestyle intervention study; made significant changes to diet/lifestyle in the past 3 months; excessive alcohol consumer (>10 standard drinks per week [33]); >5% body weight loss in the past 6 months; BMI ≥ 40 kg/m^2^ and allergic/intolerant to fig, kiwifruit or papaya. Eligible volunteers were provided with a detailed description of the study and written informed consent was obtained from all subjects involved in the study. The study protocol was approved by the Human Research Ethics Committee, University of Newcastle (H-2020-0271) and all procedures were conducted in accordance with the 1975 Declaration of Helsinki as revised in 2013. The trial was registered with the Australian New Zealand Clinical Trials Registry at https://www.anzctr.org.au/ (ACTRN 12621000190808), accessed on 11 August 2021.

### 2.2. Study Design

This study was a 12-week, double-blinded, randomised, placebo-controlled trial with two parallel groups. Volunteers were allocated to treatment groups using a computer-generated block randomisation method and stratified by sex (Random Allocation Software version 1.0.0). Participants were randomly allocated to consume one of the following dietary interventions daily for 12 weeks: 50 mL liquid drink containing either placebo (0 mg *Pinus massoniana*, providing 32 mg total polyphenols) or PMBE (1322 mg of *Pinus massoniana*, providing 432 mg total polyphenols). Participants were de-identified and assigned number codes. Both interventions were identical in sensory characteristics and were provided to participants in daily portions. Tismor Health and Wellness (Kingsgrove, NSW, Australia) manufactured and packaged both the placebo and the active products, and they were packed in individual single dose (50 mL) amber bottles with screw-top lids and looked, tasted and smelled alike. Supplement bottles and storage boxes were labelled with colour-coded stickers upon packaging by the manufacturer, and therefore, the treatment allocation could not be ascertained by the study investigators or the participants. The PMBE intervention product is commercially available as *RecoveR8*. Each daily portion of liquid provided 43.7 mL purified water, 2938.5 mg inulin, 1322 mg *Pinus massoniana*, 734.5 mg glycerin, 489.5 mg papain enzyme (derived from papaya), 171.5 mg xanthum gum, 150 mg citric acid anhydrous, 98 mg actinidia chinensis (derived from kiwifruit), 73.5 mg cranberry extract, 73.5 mg cranberry flavour and 49 mg pomegranate dry extract as the key ingredients. The placebo liquid was predominately composed of purified water, inulin, microcrystalline cellulose and small amounts of flavourings. It was devoid of *Pinus massoniana* and any other fruit extracts. The composition of polyphenolics, catechins, PACs and anthocyanosides of placebo and *RecoveR8* are presented in Table 1. Compositional analyses were conducted by an independent laboratory (Analytical Research Laboratory, Southern Cross University, Lismore, NSW, Australia). Total PACs and total polyphenolics were identified via UV spectroscopy and total catechins, total anthocyanins and procyanidins were identified through high-performance liquid chromatography and procyanidins.

Participants were instructed to consume the entire supplement each day with a main meal (preferably breakfast) as part of their habitual diet and lifestyle. Compliance was monitored by evaluation of the supplement consumption log, empty vs. full supplement bottle count-back and analysis of habitual dietary intake pre, during and post intervention.

### 2.3. Clinical Assessments

Participants attended Nutraceuticals Research Program’s clinical trial facility at the University of Newcastle (Callaghan, NSW Australia) after an overnight fast (>10 h) at baseline (0 weeks), mid-way (6 weeks) and post-intervention (12 weeks). Body weight, BMI, medical history, habitual dietary intake, physical activity patterns and fasting blood samples were collected for plasma MDA concentrations, inflammatory parameters, and liver function. Height was measured using a wall-mounted stadiometer with a movable headpiece (Seca 206 Bodymeter Wall Height Measure Ruler). Height (cm) and weight (kg) were collected to the nearest 0.1 units in light clothing without shoes.

### 2.4. Medical History, Dietary Intake and Physical Activity

A self-administered medical history questionnaire was completed by all participants at baseline to collect information regarding past and present medical conditions; history of blood lipid profile, prescribed or over-the-counter medication(s), habitual supplement use and habitual consumption of alcohol and smoking history. Habitual diet and physical activity patterns at baseline and post-intervention were assessed by a 3-day food diary and physical activity questionnaire (International Physical Activity Questionnaire; IPAQ Long Last 7 Days Self-Administered Format, October 2002), respectively. Dietary data were evaluated using FoodWorks, Professional Edition Version 10.0.4266 (Xyris^®^, Brisbane, QLD, Australia). Physical activity data were interpreted as the metabolic equivalent of task minutes per week (MET/week) to measure the energy cost of physical activities.

### 2.5. Blood Sampling and Analyses

Fasted blood samples were collected at baseline, mid-way (6 weeks) and post-intervention (12 weeks) via venepuncture into tubes pre-coated with EDTA by an experienced phlebotomist. Samples were centrifuged (Heraeus Biofuge Stratos) for 10 min at 3000× *g* at 4 °C. Plasma and red blood cell fractions were aliquoted and stored at −80 °C until further analysis. Biochemical parameters, such as fibrinogen, full blood count, liver function, and high-sensitive C-reactive protein (hsCRP) were measured on a VP auto analyser using standardised reagents by the commercial pathology service provider Pathology North.

Lipid peroxidation was measured in plasma samples using a lipid peroxidation (MDA) enzyme-linked immunosorbent assay (ELISA) kit (Abcam, Cambridge, UK) as per the manufacturer’s instructions. This method is based upon the reaction of free MDA, within the sample, with thiobarbituric acid (TBA) to generate an MDA-TBA abduct which was quantified colorimetrically using Labsystems Multiskan Ascent at 540 nm (Thermo Fisher, Waltham, MA, USA) and expressed as nmol/mL. ELISA was also used to quantify interleukin (IL) IL-6, IL-10 and intercellular adhesion molecule-1 (ICAM-1) protein levels in human serum, in duplicate, using high sensitivity human Quantikine ELISA Kits (R&D Systems, Minneapolis, MI, USA) as per manufacturer’s instructions. Assay limits ranged from 0.2 to 10 pg/mL for IL-6, 0.78 to 50 pg/mL for IL-10 and 1.6 to 50 ng/mL for ICAM-1.

### 2.6. Statistical Analysis and Sample Size Determination

Based on previous estimates of the variance in MDA concentrations in healthy older adults (Mean = 3.72, SD ± 0.7) [34] to elicit 80% power at a significance level of 0.05 to detect a 0.65 nmol/mL (~17%) difference between the placebo and PMBE group, a total of 50 participants (*n* = 25 per group) is required. To account for a potential 20% dropout rate, a total of 60 (*n* = 30 per group) participants were recruited. Allocation to treatment groups was based on a computer-generated permuted block randomisation method stratified by sex.

Data were assessed for normality using the Shapiro–Wilk test and visual plots, such as histograms and box plots. Quantitative variables were summarised using mean ± SE or median and interquartile range depending on normality. Qualitative variables were summarised by frequencies and percentages. Mean (SEM) change in MDA and other outcome measures from baseline to 6 weeks and overall (12 weeks) were summarised by placebo and PMBE group.

A mixed effect regression model was used to evaluate the mean change in participants’ MDA concentrations. The model included fixed categorial effects for elapsed time, treatment assignment and their interaction, as well as random subject-level intercept to account for within-subject correlations resulting from repeated measurements on the same participants at baseline, 6 weeks and 12 weeks. The same analyses were performed for biochemical markers of inflammation and physical activity levels. A logarithmic transformation of hsCRP was performed to reduce skewness. If models were found to be significant for change in response variables across groups, variables, such as sex, age, BMI and body fat mass age were included in the model to examine the potential effect of confounding factors. Regarding model fit, linearity and normality were assessed by graphical inspection of residuals and fitted values. All tests were two-tailed at the level of significance of 0.5 and all data were analysed using StataCorp. 2015. *Stata Statistical Software: Release 14* (College Station, TX, USA: StataCorp LP.)

## 3. Results

### 3.1. Baseline Characteristics

**Figure 1:**
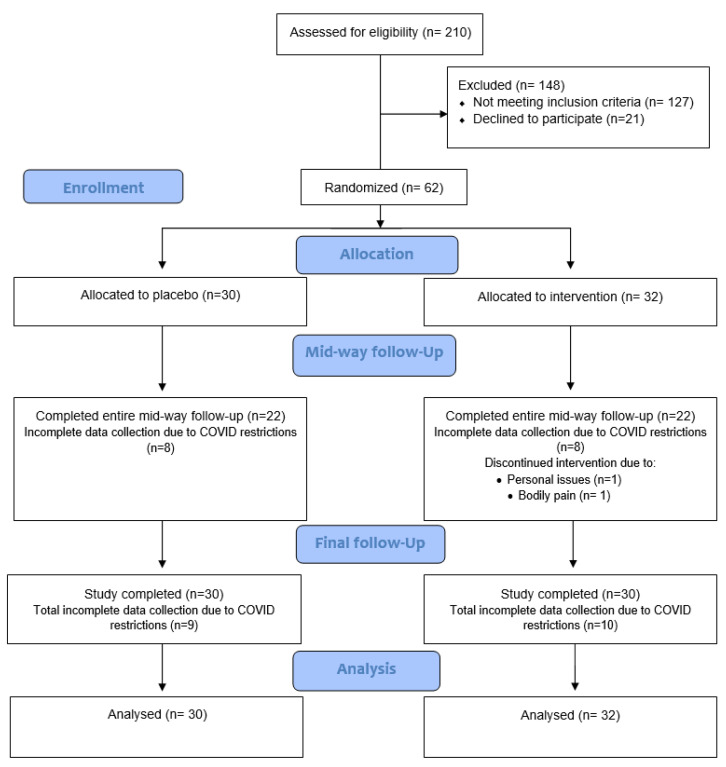
Flowchart of participant recruitment, screening, and assessment.

Sixty-two participants were recruited during the period March 2021 to mid-October 2021. Two participants dropped out of the trial due to undisclosed personal reasons (*n* = 1) and bodily pain (*n* = 1). Due to NSW Health Government restrictions in response to the COVID-19 pandemic, a lockdown in the Hunter area occurred between early August to early October which resulted in incomplete data collection for eight participants in both the placebo group and PMBE group who were due for a 6-week follow-up timepoint. A further one participant from the placebo group and two participants from the PMBE group had incomplete data collected at 12 weeks. A total of 60 participants completed the trial and all available data from participants randomised from baseline were included in the final analysis (*n* = 62), Figure 1. Most participants were female (60%) and identified as Oceanian (Australian) ethnicity (52%) with a mean age of 64.6 ± 5.1 years, BMI of 25.2 ± 3.2 kg/m^2^, waist circumference 87.3 ± 10.2 cm (females) and 98.2 ± 9.4 cm (males). Only 15% of individuals were taking medications for blood pressure, 10% for high cholesterol, 10% for gastrointestinal reflux issues, 8% for anxiety and 21% for other various conditions. Following randomisation, the placebo group and PMBE group were comparable at baseline and did not significantly differ in characteristics, Table 2.

### 3.2. Nutrient Intake, Physical Activity and Compliance

Groups were similar at baseline for nutrient intake parameters, Table 3. No statistically significant changes in nutrient intake within groups were evident from baseline. Mean change in nutrient intake parameters from baseline did not statistically significantly differ across groups. The study supplement was well tolerated by participants with excellent compliance overall (98.7 ± 2.3%) which was comparable across both groups, Table 2. Dietary intake of antioxidants compounds was similar across groups and did not significantly alter across timepoints, except for vitamin E intake which was significantly reduced at 12 weeks compared to baseline in the placebo group only (Supplementary Table S1). Mean change in dietary antioxidant compounds from baseline to 12 weeks was not significantly different between groups. Physical activity did not significantly change from baseline to post-intervention within and between groups.

### 3.3. Anthropometry and Physical Activity

Body weight and BMI remained the same both within and across groups throughout the duration of the trial. Physical activity did not significantly alter within groups across study timepoints, nor did change in physical activity levels differ across groups at any timepoints (Supplementary Table S2).

### 3.4. Primary Outcome: Plasma MDA Concentrations

**Figure 2:**
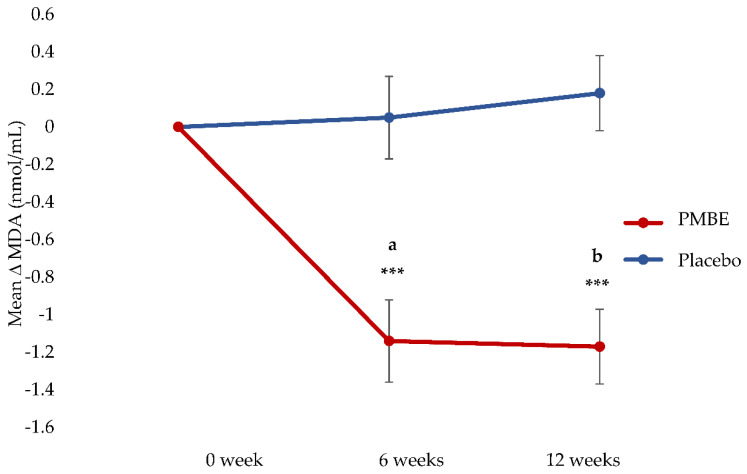
Mean change in MDA concentrations (with standard error bars) over time in the placebo and PMBE groups. Significant change from baseline is indicated by *** *p* < 0.0001; (**a**), mean change between PMBE group and placebo group at 6 weeks was statistically significant (−1.23 nmol/mL, 95% CI −1.84 to −0.62, *p* < 0.0001). (**b**), mean change between PMBE group and placebo group at 12 weeks was statistically significant (−1.53 nmol/mL, 95% CI −2.09 to −0.98, *p* < 0.0001).

The mean change in MDA concentrations at each time point in the PMBE group was significantly different from the mean change in MDA concentrations in the placebo group at each timepoint (−1.23 nmol/L at 6 weeks and −1.53 nmol/L at 12 weeks; *p* < 0.0001), Table 4. Compared to baseline, MDA concentrations significantly reduced at 6 weeks (−1.19 nmol/mL) and 12 weeks (−1.35 nmol/mL) in the PMBE group. Changes in the MDA concentrations in the placebo group were not significantly different at 6 weeks or 12 weeks when compared to baseline, Figure 2.

### 3.5. Inflammatory Markers

The difference in the change in IL-6 concentrations from baseline to 12 weeks between the placebo group and PMBE group was significantly different, such that the PMBE group increased by 0.30 pg/dL (*p* = 0.035) compared to the placebo (Table 4). There were no significant changes within either group over time. Fibrinogen concentrations were significantly reduced in the PMBE group (−0.25 g/L, *p* < 0.0001) at 12 weeks compared to baseline. There were no significant changes in other inflammatory markers (hsCRP, IL-10 and ICAM-1).

### 3.6. Liver Function

Liver function as an indicator of safety monitoring remained stable throughout the trial and outcomes remained similar within groups, Table 5. No significant changes were observed in liver function parameters, except for a slight rise in alkaline phosphatase concentrations (+2.8 U/L) at 12 weeks when compared to baseline in the PMBE group only. Liver function tests remained in the normal reference targets at all study timepoints in both groups.

## 4. Discussion

Our findings demonstrate that daily dietary supplementation with a polyphenol-rich drink derived from PMBE for 12 weeks significantly reduced oxidative stress as indicated by a 14% reduction in MDA concentrations in free-living healthy older adults. Concurrently, fibrinogen concentrations were also significantly reduced following 12-week dietary supplementation with PMBE compared to baseline. Compared to placebo; a rise in IL-6 concentrations was observed at 12 weeks in the PMBE group. This is the first study to investigate the physiological effects of polyphenols derived from PMBE on oxidative stress in humans.

The daily provision of 435 mg of polyphenols in the PMBE supplement in the current study is significant given daily dietary intakes of 280–300 mg/d have been previously reported from 24-h recalls among countries, such as Australia [36], the USA and Spain [37]. Polyphenol intake for the background diet was not measured in the current study; however, nutrient intake remained stable within each group for the duration of the study and groups did not significantly differ in food group intake or nutrient status. Polyphenols delivered by various foods, such as green tea and berries have been shown to lower MDA concentrations in humans [38], and a significant reduction in oxidative stress indicated by oxygen radical absorbance capacity (ORAC) was reported in humans supplemented with 150 mg/day polyphenol-rich French maritime pine bark extract for 6 weeks [39]. Thus, the additional 50–100% polyphenols supplemented on top of polyphenols acquired by the background diet is likely a key contributor to the reduction in MDA concentrations reported in the PMBE group in this study. In the current study, key quantifiable compounds, such as polyphenolics, catechins, proanthocyanidins and anthocyanosides were measured and reported by an independent external laboratory. It is indeed likely that condensed tannins and other components (lignins, etc.) could make up the remaining difference. This is a proof-of-concept study, the first using this formulation, prompting future studies to further explore the physiological effects of these bioactive components.

Preclinical evidence demonstrates MDA-modulating effects following the administration of specific polyphenolic compounds. Procyanidins have been shown to protect in vivo cellular oxidative damage by significantly lowering MDA concentrations [40]. A study in rats with methotrexate-induced oxidative stress reported significantly lower MDA concentrations in rats randomised to 100 mg/kg oral PAC gavage for 4 days [41]. SOD and glutathione peroxidase levels were also increased and jejunal damage was decreased in these rats; suggesting PACs may protect the small intestine of rats from oxidative stress induced by methotrexate as a result of its potent antioxidant properties [41]. Similar findings were evident in rats with myocardial ischaemic injury treated with 100 mg/kg PACs from grape seed extract twice daily by oral gavage for 3 weeks, whereby significantly lower MDA levels were reported in these rats compared to untreated rats [42]. SOD was also significantly higher in PAC-treated rats compared to untreated rats. Cholesterol-fed rabbits supplemented with PAC-rich extracts from grape seed had significantly decreased aortic MDA content compared to rabbits with feed not enriched with PACs. In addition, a reduced number of oxidised LDL-positive macrophage-derived foam cells in atherosclerotic lesions of rabbit aortas was also detected after feeding PAC-rich extract; resulting in significantly reduced severe atherosclerosis in the aorta [43].

Limited human evidence exists regarding the effects of specific polyphenolic compounds, such as PACs and procyanidins on MDA concentrations. Healthy humans with mildly elevated low-density lipoprotein cholesterol (LDL-C) supplemented 200 mg/day or 400 mg/day grape seed extract (calculated as PACs) tablets had significantly reduced MDA-modified LDL-C after 12 weeks compared to the placebo group. These findings suggest an attenuation of LDL-C oxidation, with potential useful application in preventing lifestyle-related diseases, such as atherosclerosis [44]. The antioxidant properties of catechins derived from tea have been shown in a human in vitro study to lower MDA levels in erythrocytes and lower glutathione oxidation [45]. Thus, it is likely that the combination of polyphenols, such as catechins and PACs in our study has had a combined effect on lowering MDA concentrations. Further exploration into the absorption, distribution, metabolism and excretion of catechins to ascertain the species-dependent (i.e., humans vs. animal) differences in the metabolism and subsequent effects on markers of oxidative stress are warranted [46].

The combination of polyphenol-rich ingredients in the PMBE supplement used in the current study may also provide synergy between the various plant bioactives for not only greater delivery of polyphenols, but potentially enhanced bioavailability and absorption. Papain derived from papaya and Actinidia chinensis (actinidin) derived from golden kiwifruit are known proteolytic enzymes [47,48]. The presence of these enzymes in the intervention formulation may play a role in aiding protein breakdown during digestion and may promote absorption and solubility of PMBE. Golden kiwifruit supplementation in a Western diet for 8 weeks has been previously shown to significantly reduce oxidative damage in humans [49] and papain has been shown to inhibit lipid peroxidation to a similar extent to vitamin C and vitamin E [47,50]. Thus, the combination of these bioactives along with PMBE in the dietary supplement may provide complementary and/or synergistic effects; enhancing the overall efficacy of the intervention to yield antioxidant effects in humans.

Oxidative stress and inflammation are two closely interrelated and interdependent pathophysiological processes that play an important role in ageing [51] and age-related diseases, such as cardiovascular disease [52,53], type 2 diabetes [54], cancer [55] and neurodegenerative diseases [56]. The significantly lower fibrinogen levels following PMBE supplementation in the current study indicate decreased inflammatory processes in the body. Fibrinogen being an acute phase protein, reflects systemic inflammatory processes [57]. Fibrinogen levels have been lowered in osteoarthritis patients treated with 100 mg/day French maritime pine bark extract [58]. Whereas participants in the current study population had normal fibrinogen levels and thus provide novel findings for the potential implications of the PMBE supplement as a systemic anti-inflammatory agent. Conversely, placebo individuals in the PMBE group also had a statistically significant rise in IL-6 concentrations, this is unexplainable; the individuals’ baseline IL-6 was low-normal and despite rising, remained in the healthy-normal range. An animal study recently reported reductions in serum pro-inflammatory mediators, such as IL-6, IL-1β and tumour necrosis factor-α and increases in the anti-inflammatory cytokine IL-10 following PMBE supplementation [59]; however, there are currently no studies investigating the anti-inflammatory capacity of PMBE in humans. Future studies powered to detect clinically relevant changes in inflammatory mediators following treatment with PMBE extract formulations in humans are warranted to elucidate their impact on systematic inflammation.

This study is the first study to explore the physiological effects of a polyphenol-rich dietary supplement derived from PMBE combined with other plant bioactives for reducing oxidative stress. It is also the first study to demonstrate the safety of this specific polyphenol-rich supplement in humans (Table 5). Future studies with a placebo comparison that is identical but void of PMBE would allow for distinction between the degree of complementarity and/or synergy occurring between PMBE and the other bioactives (i.e., papain, Actinidia chinensis, cranberry extract, etc.). A limitation of the study is that some data for participants at the follow-up timepoints were missing for secondary outcomes; however, this was unavoidable due to the state government public health orders associated with the COVID-19 pandemic at the time. The use of mixed effect regression was intentional because the test automatically handles missing data in the outcome under the missing at random assumption. Polyphenols provided by the participant’s habitual diets may have influenced study findings; however, this was not quantified as few phytochemical-specific food composition databases exist globally [60]. Moreover, database scarcity combined with known issues around utilising food composition databases that are non-specific to geographical location makes accurate quantification of dietary polyphenol intake a challenge [60]. Nonetheless, we have demonstrated that the antioxidant capacity of PMBE in this study was independent of background dietary intake of micronutrients with known antioxidant properties, which did not change during the study (Supplementary Table S1). Future studies are warranted to explore the other markers of oxidative stress, such as oxo-histidine and 8-hydroxyguanine, to explore the influence of these bioactives on protein and DNA oxidation. The double-blinded randomised study design, excellent compliance, demonstration of product safety, adequately powered sample size and use of a high-quality manufactured dietary supplement are key strengths of this study. Participants were also free-living community-dwelling individuals who maintained their habitual diet and lifestyle whilst participating in the study, yet compliance was excellent; therefore, our findings are highly transferrable to the healthy older Australian population. Finally, the study duration was adequate to demonstrate a modulation in human plasma MDA concentrations. Future long-term studies in diseased populations would substantiate our findings as well as provide insight into the other health-promoting/disease risk-reducing capabilities of this polyphenol-rich dietary supplement derived from PMBE.

## 5. Conclusions

Findings from this study provide novel evidence for the use of a polyphenol-rich dietary supplement derived from PMBE and fruit bioactives for lowering MDA concentrations in healthy ageing adults. These findings support the investigation of this dietary supplement in other disease applications where oxidative stress is heightened, e.g., atherosclerosis, osteoarthritis, or metabolic syndrome. This study also provides evidence for the safety of this product for human consumption for 12 weeks (Table 5) in addition to the habitual diet and lifestyle of community-dwelling individuals. Moreover, clinical measurements and usual diet and physical activity regimes did not change and thus demonstrate a practical and translational solution for alleviating some ageing-induced oxidative stress. Although not explored in this paper, this intervention has potential applications for blood-pressure lowering [61], positively impacting metabolic diseases [62] and alleviating muscle cramps and pain [63]; consistent with clinical findings utilising other types of pine bark extracts. The impact on inflammatory status requires further investigation in an adequately powered study, given the bidirectional relationship between oxidative stress and systemic inflammation. Findings from this study could provide an effective therapeutic strategy for supporting the ageing process; potentially mitigating age-associated metabolic dysfunction that is underpinned by heightened oxidative stress and raised systemic inflammation. Further research is warranted to investigate the non-oxidative modulating roles this polyphenol-rich dietary supplement has to offer, as well as to explore the optimal dose, timing and duration for optimal health outcomes.

## Figures and Tables

**Table 1:** Composition of study products ^1^.

	Placebo (mg/Day)	PMBE (mg/Day)
Polyphenolics (CE)	32.0	431.5
Monomeric (free) catechins	2	29.5
Total PACs	10.5	59.5
Total anthocyanosides	nd	0.25

^1^ Composition is reported for mg per daily dose (50 mL) of study product. Composition was quantified by The Analytical Research Laboratory at Southern Cross University. CE, catechin equivalents; nd, not detected; PACs, proanthoycanidins.

**Table 2:** Participant characteristics at baseline in the placebo and PMBE groups ^1^.

	Placebo (*n* = 30)	PMBE (*n* = 32)	*p*
Sex, *n* (%)			0.960
Male	12 (40.0)	13 (40.6)	
Female	18 (60.0)	19 (59.4)	
Ethnicity, *n* (%)			0.681
Oceanian	17 (56.7)	15 (46.9)	
Oceanian/North-west European	5 (16.7)	6 (18.8)	
North-west European	5 (16.7)	9 (28.1)	
South-east European	1 (3.3)	0 (0)	
Other ^2^	2 (6.7)	2 (6.3)	
Age (y)	63.85 ± 0.92	65.22 ± 0.90	0.293
Height (cm)	166.90 ± 1.68	166.7 ± 1.78	0.941
Weight (kg)	71.50 ± 2.36	70.83 ± 2.64	0.851
BMI (kg/m^2^)	25.33 ± 0.58	25.06 ± 0.59	0.746
Medication use for:			
High blood pressure	4 (13.3)	5 (15.6)	0.798
High cholesterol	1 (3.3)	5 (15.6)	0.102
GORD	2 (6.7)	4 (12.5)	0.438
Anxiety	4 (13.3)	1 (3.1)	0.140
Other ^3^	8 (26.7)	5 (15.6)	0.286
MET (min/week) ^4^	4329.6 (2493, 6456)	4452 (1983, 6698)	0.822
Compliance ^5^	98.85 ± 0.52	98.61 ± 0.31	0.596

^1^ Values are reported as means ± SEM or median or interquartile range (25th percentile, 75th percentile) depending on data distribution for continuous measures and as *n* (%) for categorical measures. Independent samples *t*-test was used to compare baseline data across groups for normally distributed continuous data, Wilcoxon rank-sum test for non-normally distributed data and chi-square for categorical data. ^2^ Other races include South-East Asian (*n* = 1), Oceanian/Southern and Eastern European (*n* = 1), North-West European/North African and Middle Eastern (*n* = 1) and North African and Middle Eastern/Sub-Saharan African (*n* = 1). ^3^ Values reported as median and (interquartile range) as data is non-normally distributed. ^4^ Other includes medications for hypothyroidism, herpes, hormone replacement. ^5^ Compliance is reported for all 60 participants who completed the 12-week intervention. GORD, gastroesophageal reflux disease; MET, metabolic equivalent; PMBE, *Pinus massoniana* bark extract; PL, placebo.

**Table 3:** Background nutrient intake in the placebo and PMBE group at baseline, 6 weeks and 12 weeks (post-intervention) and mean change from baseline to 12 weeks post-intervention (∆) ^1^.

	Time			∆
Nutrients	Baseline	6-Weeks	12-Weeks	
Energy (kJ)				
Placebo	8554.9 (391.5)	8489.3 (373.6)	8452.4 (421.1)	−102.4 (327.3)
PMBE	8307.1 (359.2)	8364.8 (434.8)	8689.8 (356.8)	371.3 (318.8)
Protein (%E) ^2^				
Placebo	18.8 (0.8)	19.0 (0.9)	19.1 (0.9)	0.2 (0.9)
PMBE	18.7 (0.5)	17.4 (0.8)	17.5 (0.7)	−1.2 (0.6)
CHO (%E)				
Placebo	39.1 (1.4)	38.3 (1.4)	39.1 (1.6)	0.08 (1.3)
PMBE	39.3 (1.4)	39.9 (1.3)	40.0 (1.3)	1.0 (1.4)
Fat (%E)				
Placebo	36.1 (1.3)	35.9 (1.5)	35.7 (1.5)	−0.5 (1.3)
PMBE	36.0 (1.1)	35.2 (1.1)	35.4 (0.9)	−0.7 (1.2)
Sat Fat (%E)				
Placebo	13.0 (0.7)	13.3 (0.8)	13.2 (0.8)	0.2 (0.8)
PMBE	13.5 (0.6)	13.0 (0.6)	13.1 (0.5)	−0.5 (0.6)
Trans fat (%E)				
Placebo	0.6 (0.08)	0.5 (0.05)	0.6 (0.04)	−0.04 (0.08)
PMBE	0.6 (0.04)	0.6 (0.04)	0.6 (0.04)	−0.05 (0.05)
Cholesterol (mg)				
Placebo	328.1 (41.1)	288.7 (31.6)	306.3 (27.5)	−21.8 (42.9)
PMBE	363.3 (26.9)	288.4 (29.3)	314.7 (26.3)	−32.9 (27.7)
Sugars (g)				
Placebo	91.6 (6.9)	91.3 (5.2)	90.4 (5.7)	−1.2 (6.3)
PMBE	90.9 (6.3)	93.3 (7.0)	93.7 (6.8)	3.5 (6.1)
Fibre (g)				
Placebo	29.0 (1.9)	28.9 (1.9)	29.1 (2.4)	0.06 (1.5)
PMBE	28.0 (1.6)	27.6 (1.5)	28.2 (1.5)	0.2 (1.7)
Sodium (mg)				
Placebo	2417.9 (164.3)	1984.1 (116.4)	2094.7 (133.3)	−323.1 (197.4)
PMBE	2365.9 (118.8)	2247.3 (167.9)	2520.6 (192.3)	174.8 (213.5)
Alcohol (g)				
Placebo	7.6 (2.8)	9.9 (3.0)	8.4 (2.3)	0.8 (1.5)
PMBE	7.7 (1.8)	10.9 (2.5)	10.5 (1.9)	2.3 (2.0)

Dietary data from 3-day food diaries were evaluated using FoodWorks, Xyris^®^, Professional Edition Version 10.0.4266. Values are reported as mean (SEM). Independent samples *t*-test was used to compare baseline data and mean change data across groups. Paired samples *t*-test was used to compare change from baseline to post-intervention within groups. ^1^ Baseline data are for all participants who commenced the trial (*n* = 30 placebo, *n* = 32 intervention). For the active group, data at 6 weeks represent 31 participants and data at 12 weeks represent 30 participants who remained in the trial at the timepoints. ^2^ Data are presented for participants who completed the trial (*N* = 60). CHO, carbohydrates; PMBE, *Pinus massoniana* bark extract; %E refers to the percentage of dietary energy that is contributed by the relevant macronutrient; ∆, change from baseline to post-intervention.

**Table 4:** Biochemical parameters in the placebo group and PMBE group at baseline, 6 weeks and 12 weeks (post-intervention) and changes in biochemical parameters from baseline at 6 weeks and 12 weeks ^1^.

	Time					Change ^2^	
Outcomes	Baseline	6-Weeks	*n*	12-Weeks	*n*	∆1 (95% CI) ^2^	∆2 (95% CI) ^3^
MDA (nmol/mL)							
Placebo	7.58 (0.12)	7.57 (0.12)	22	7.75 (0.14)		0.05 (−0.39, −0.48)	0.18 (−0.22, 0.57)
PMBE	8.23 (0.19)	7.02 (0.27)	22	6.85 (0.14)	30	−1.19 (−1.62, −0.75) ***	−1.35 (−1.74, −0.96) ***
*Difference* ^4^						−1.23 (−1.84, −0.62) ***	−1.53 (−2.09, −0.98) ***
hsCRP (mg/dL) ^5^							
Placebo	1.26 (0.71. 2.21)	1.44 (0.89, 2.45)	22	1.43 (0.86, 3.22)		0.01 (−0.23, 0.26)	0.18 (−0.04, 0.40)
PMBE	0.92 (0.53, 1.72)	0.68 (0.42, 1.34)	22	0.75 (0.49, 1.36)	30	−0.20 (−0.44, 0.05)	−0.10 (−0.32, 0.12)
*Difference*						−0.21 (−0.56, 0.14)	−0.28 (−0.59, 0.03)
IL-6 (pg/mL)							
Placebo	1.00 (0.63, 1.56)	0.85 (0.58, 1.27)	22	0.83 (0.58, 1.27)		−0.21 (−0.44, 0.01)	−0.13 (−0.33, 0.07)
PMBE	0.93 (0.51, 1.41)	0.91 (0.65, 1.19)	22	1.04 (0.62, 1.46)	30	0.05 (−0.18, 0.27)	0.17 (−0.03, 0.37)
*Difference*						0.26 (−0.06, 0.58)	0.30 (0.02, 0.59) *
IL-10 (pg/mL)							
Placebo	0.49 (0.23, 1.21)	0.65 (0.29, 1.26)	22	0.65 (0.38, 0.97)		0.12 (−0.09, 0.34)	0.02 (−0.19, 0.23)
PMBE	1.13 (1.06, 1.18)	1.17 (1.03, 1.22)	22	1.16 (1.11, 1.31)	30	0.01 (−0.03, 0.06)	0.02 (−0.03, 0.08)
*Difference*						−0.11 (−0.33, 0.10)	0.001 (−0.22, 0.22)
ICAM-1 (ng/mL)							
Placebo	11.66 (0.35)	11.67 (0.46)	22	11.80 (0.41)		−0.23 (−0.71, 0.25)	0.16 (−0.26, 0.59)
PMBE	11.03 (0.46)	11.80 (0.49)	22	11.13 (0.53)	30	0.11 (−0.36, 0.57)	0.12 (−0.23, 0.47)
*Difference*						0.34 (−0.34, 1.01)	−0.04 (−0.64, 0.56)
Fibrinogen (g/L)							
Placebo	3.56 (0.09)	3.55 (0.12)	22	3.37 (0.11)		−0.05 (−0.19, 0.09)	−0.18 (−0.38, −0.009)
PMBE	3.17 (0.08)	3.22 (0.08)	22	2.90 (0.09)	30	−0.02 (−0.14, 0.10)	−0.25 (−0.39, −0.11) ***
*Difference*						0.03 (−0.16, 0.21)	−0.07 (−0.31, 0.17)

^1^ Baseline data are for all participants who commenced the trial (*n* = 30 placebo, *n* = 32 active). Data at 6 and 12 weeks are presented for all participants unless otherwise specified in respective table columns and presented as mean (SEM) or median or interquartile range (25th percentile, 75th percentile) depending on data distribution. Mixed models were used to examine the effect of time within treatment groups as well as the interaction between time and treatment across groups. Data for mixed models are presented as mean estimates (95% confidence intervals). All data presented are for adjusted models only using pre-specified variables. Significant findings are indicated * *p* < 0.05 and *** *p* < 0.0001. ^2^ Effect of time within treatment group from baseline to 6 weeks. ^3^ Effect of time within treatment group from baseline to 12 weeks (post-intervention). ^4^ Interaction between time x treatment is presented as intervention minus control. ^5^ Data were log-transformed because the model did not fulfill the assumptions for mixed linear modelling. hsCRP, high sensitive C-Reactive protein; ICAM-1, intercellular adhesion molecule-1; IL, interleukin; MDA, malondialdehyde; PMBE, *Pinus massoniana* bark extract.

**Table 5:** Liver function parameters at baseline, 6 weeks and post-intervention in the placebo and PMBE groups.

	Time			Reference Intervals ^1^
Outcomes	Baseline	6-Weeks	12-Weeks	
Calc Glob (g/L)				
Placebo	28.73 (0.36)	28.64 (0.38)	29.07 (0.42)	25–35
PMBE	29.59 (0.50)	29.68 (0.62)	29.50 (0.56)	
T. Bilirubin (umol/L)				
Placebo	10 (8, 12)	9 (7, 12)	10 (9, 13)	<20
PMBE	13 (9, 19)	12 (11, 20)	13 (8, 23)	
GGT (U/L)				
Placebo	16 (13, 21)	17.5 (13, 24)	17.5 (13, 26)	<50 (male)
PMBE	18 (13, 24.5)	17.5 (14, 27)	17.5 (14. 24)	<30 (female)
ALP (U/L)				
Placebo	74.10 (2.82)	77.14 (2.53)	74.83 (3.15)	30–110
PMBE	65.44 (2.62)	67.95 (3.41)	68.00 (3.19) *	
ALT (U/L)				
Placebo	23.5 (20, 32)	25 (22, 33)	24 (21, 30)	<35
PMBE	23 (21, 27.5)	24.5 (21, 30)	24.5 (20, 30)	
AST (U/L)				
Placebo	26 (23, 29)	26 (24, 29)	25.5 (24, 29)	<40
PMBE	25 (23, 30)	26 (24, 29)	25 (22, 29)	

Values are reported as mean (SEM) or median (25th percentile, 75th percentile) depending on distribution of data. Paired *t*-test was used to compare changes from baseline to post-intervention for normally distributed data and Wilcoxon signed rank test for non-normally distributed data within groups. * *p* < 0.05. ^1^ Reference Intervals for Liver Function Tests by The Royal College of Pathologists of Australia (RCPA) [35]. ALP, alkaline phosphatase; ALT, alanine aminotransferase; AST, aspartate transaminase; Calc Glob, calculated globulin; PMBE, *Pinus massoniana* bark extract; T. Bilirubin, total bilirubin.

## Data Availability

The data presented in this study are available in the article and Supplementary Materials.

## Supplementary Materials

The following supporting information can be downloaded at: https://www.mdpi.com/article/10.3390/antiox11081560/s1, Table S1: Background dietary intake of antioxidant compounds in the placebo and PMBE group at baseline, 6 weeks and 12 weeks (post-intervention) and mean change from baseline to post-intervention (∆); Table S2: Change in body weight, BMI and physical activity levels in the placebo group and PMBE group from baseline, 6-weeks and 12-weeks (post-intervention).
