# Supplementary material for: Antioxidant Effects of a Polyphenol-Rich Dietary Supplement Incorporating Pinus massoniana Bark Extract in Healthy Older Adults: A Two-Arm, Parallel Group, Randomized Placebo-Controlled Trial
Source: Antioxidants (Basel). 2022 Aug 11;11(8):1560. doi: 10.3390/antiox11081560 (PMC9405161; doi:10.3390/antiox11081560)
Supplement: Supplementary file 1 [file antioxidants-11-01560-s001.zip › antioxidants-1813852-supplementary.pdf]

Supplementary Table S1. Background dietary intake of antioxidant compounds in the placebo and PMBE group at baseline, 6-weeks and 12-weeks (post-intervention) and mean change from baseline to post-intervention (Δ).[1]

	Time			Δ
**Nutrients**	**Baseline**	**6-weeks**	**12-weeks**	
Vitamin C (mg)				
Placebo	97.4 (67.1, 129.6)	79.4 (54.8, 136.9)	82.2 (50.3, 118.9)	−4.4 (−47.1, 54.2)
PMBE	97.9 (46.25, 150.1)	95.6 (60.4, 147.8)	118.5 (80.2, 135.8)	26.5 (−32.1, 75.0)
Vitamin E (mg)				
Placebo	14.3 (9.7, 16.3)	11.7 (8.4, 15.6)	11.8 (7.3, 14.8)*	−2.3 (−6.5, −0.6)
PMBE	11.3 (9.6, 17.5)	12.9 (8.6, 17.6)	12.6 (9.7, 15.8)	−0.03 (−3.5, 3.0)
Retinol (µg)				
Placebo	234.1 (181.6, 409.5)	246.7 (172.8, 388.1)	265.1 (177.8, 451.5)	40.8 (−119.1, 137.1)
PMBE	266.0 (182.3, 354.9)	211.2 (161.8, 451.2)	305.7 (208.2, 406.2)	−10.3 (−101.7, 43.3)
Beta-carotene (µg)				
Placebo	3923.8 (2098.5, 6933.7)	4070.6 (1838.1, 6552.3)	4030.6 (2277.8, 6032.8)	247 (−2855.8, 2632.1)
PMBE	3539.0 (1659.8, 6213.4)	3422.0 (1696.8, 7201.1)	4135.2 (2859.6, 6348.4)	219.5 (−1791.7, 2254.9)
Zinc (mg)				
Placebo	10.2 (8.0, 12.3)	9.9 (8.9, 12.7)	9.4 (8.4, 14.0)	0.4 (−2.0, 1.9)
PMBE	10.7 (7.8, 13.5)	9.1 (7.8, 11.1)	9.9 (8.4, 11.4)	−0.6 (−2.6, 2.2)
Selenium (µg)				
Placebo	78.5 (60.0, 96.6)	74.6 (59.2, 91.8)	84.8 (61.3, 99.2)	−0.1 (−20.5, 9.8)
PMBE	82.4 (59.7, 104.5)	74.0 (53.6, 103.1)	68.3 (58.4, 92.5)	−3.8 (−24.9, 13.1)

Values are reported as median (25th percentile, 75th percentile) as data was non-normally distributed. Wilcoxon signed-rank test was used to compare change from baseline to post-intervention within groups. Kruskal-Wallis rank test was used to compare mean change from baseline to post-intervention across groups. $^{*}p < 0.05$

[1] Baseline data is for all participants who commenced the trial (n = 30 placebo, n = 32 intervention). For the active group, data at 6 weeks represents 31 participants and data at 12 weeks represents 30 participants who remained in the trial at the timepoints.

[2] Data is presented for participants who completed the trial (N = 60)

PMBE, *Pinus massoniana* bark extract.

Supplementary Table S2. Change in body weight, BMI and physical activity levels in the placebo group and PMBE group from baseline, 6-weeks and 12-weeks (post-intervention).[1]

	Time					Change	
**Outcomes**	**Baseline**	**6-weeks**	***n***	**12-weeks**	***n***	**Δ 1 (95% CI)[2]**	**Δ 2 (95% CI)[3]**
Weight (kg)							
Placebo	71.50 (2.36)	71.71 (2.55)	29	71.96 (2.49)		−0.09 (−0.65, 0.48)	0.48 (−0.07, 1.04)
PMBE	70.83 (2.64)	69.89 (2.66)	29	69.80 (2.60)	30	0.04 (−0.53, 0.60)	0.29 (−0.28, 0.86)
Difference						0.12 (−0.68, 0.92)	0.20 (−1.00, 0.60)
BMI (kg/m^2)							
Placebo	25.33 (0.58)	25.42 (0.62)	29	25.39 (0.62)		−0.09 (−0.29, 0.12)	−0.08 (−0.13, 0.28)
PMBE	25.06 (0.59)	24.80 (0.57)	29	24.75 (0.55)	30	0.04 (−0.16, 0.25)	0.06 (−0.14, 0.27)
Difference						0.13 (−0.16, 0.42)	−0.01 (−0.30, 0.28)
MET (mins/week)							
Placebo	4329.6 (2493, 6456)	3685.5 (2547, 5595)		4452 (1983, 6698)		−593 (−1717, 532)	170 (−942, 1282)
PMBE	3889.5 (2388, 7425)	4065 (1836, 6355)	31	4545 (2961, 6132)	29	−379 (−1496, 737)	−28 (−1145, 1088)

Difference		213 (−1371, 1798)	−198 (−1774, 1378)

[1] Baseline data is for all participants who commenced the trial (*n* = 30 placebo, *n* = 32 active). Data at 6 and 12 weeks is presented for all participants unless otherwise specified in respective table columns and presented as mean (SEM) or median or interquartile range (25th percentile, 75th percentile) depending on data distribution. Mixed models were used to examine the effect of time within treatment groups as well as the interaction between time and treatment across groups. Data for mixed models is presented as mean estimates (95% confidence intervals). All data presented is for adjusted models only using pre-specified variables.

[2] Effect of time within treatment group from baseline to 6 weeks

[3] Effect of time within treatment group from baseline to 12 weeks (post-intervention)

[4] Interaction between time x treatment is presented as intervention minus control.

MET, metabolic equivalent; PMBE, *Pinus massoniana* bark extract;
